# Editors and Authors of Medical Journals Should Be Mindful of the New ICMJE Disclosure Form and Updated Policies

**DOI:** 10.31662/jmaj.2023-0088

**Published:** 2023-10-05

**Authors:** Jaime A. Teixeira da Silva, Timothy Daly

**Affiliations:** 1 Independent researcher, Kagawa, Japan; 2 Bioethics Program, FLACSO Argentina, Buenos Aires, Argentina; 3 Science Norms Democracy UMR 8011, Sorbonne Université, Paris, France

**Keywords:** accountability, ethics, conflicts of interest, editorial responsibility, transparency

## Abstract

The International Committee of Medical Journal Editors (ICMJE) recommendations are used by medical journals worldwide to guide editors and authors regarding “best practices” related to the intersection between research and publishing. In this opinion paper, we bring two discussion points to the attention of readers and users of the ICMJE recommendations. The first pertains to journals’ use of the old conflicts of interest form, replaced in 2021 with a new disclosure form. The second relates to inconsistent or outdated policies in journals’ instructions for authors mismatching the current ICMJE recommendations. The ICMJE does not monitor how journals use or apply the ICMJE recommendations. Thus, the editors must be mindful of updates and changes relevant to the authors. Furthermore, authors should carefully examine journals before submission to ensure that journals use updated forms and policies and should be mindful of submitting to non-ICMJE-recommendations-conforming journals despite claiming to follow them.

The International Committee of Medical Journal Editors (ICMJE) “Recommendations for the Conduct, Reporting, Editing, and Publication of Scholarly Work in Medical Journals,” or the ICMJE recommendations in short, are used by medical journals worldwide to guide their editors and authors regarding “best practices” related to research and publishing. Like most guidelines existing for journals, editors, and academics, their content is rightfully evolving, and these guidelines are updated to either reflect new challenges in the world of publishing ethics or new policies adapting new philosophies emerging from the research environment.

The *Japan Medical Association Journal* (*JMAJ*) claims to use the ICMJE recommendations and requests that authors abide by them. However, some of the requirements can be challenging to meet and might reflect cultural limitations. For example, a recent opinion paper in *JMAJ* highlights challenges that residency specialists face, namely when publishing papers, one of the criticisms being that complex (i.e., difficult to achieve or obtain) permissions are required to make the secondary publication of papers originally published in Japanese compliant with the ICMJE recommendations ^(1)^.

The ICMJE recommendations are refined every year or two, possibly in response to academics’ concerns made about them ^(2)^. For example, in that paper, it was questioned whether those journals that claimed to follow the ICMJE recommendations were evidently doing so. Thus, a journal merely claiming to follow these guidelines does not necessarily imply that such is being followed. Advice had been offered by the ICMJE for authors to avoid citing articles in predatory or pseudojournals, yet the ICMJE offered no such list. How can authors confidently know whether a paper belongs to a valid (scholarly) or a predatory journal? Moreover, should the ICMJE dictate what research academics can or cannot cite, and why would such a policy be nondiscriminatory and not a violation of authors’ rights and freedoms to cite whatever literature they feel is important? Consequently, while some recommendations by the ICMJE may look “good” and valid on paper, as their wording is adjusted to a fine scale with each “update,” some are unrealistic in practice. This suggests that despite the long history of the ICMJE recommendations, they are still lacking and are thus imperfect. Finally, that paper highlighted the “unfair” privilege that the ICMJE has in publishing multiple copies of its recommendations across as many journals as it deems fit or as wide as its influential network spreads. This is a luxury that average academics do not have.

As another very prominent example of the fluid nature of changes to the ICMJE recommendations, many medical journals rely on them to define authorship, requesting authors to make statements validating their authorship and contribution to the stated research. However, making a statement on paper is one thing, but verifying the validity of authorship is a completely different issue. Thus, as argued above, some of the ICMJE recommendations sound good in principle, but in practice, they are extremely difficult to implement, especially authorship verification. In journals claiming to follow the ICMJE recommendations, authors must satisfy four clauses to meet the ICMJE’s definition of authorship. However, historically, this was rarely the case. The ICMJE authorship clauses were yesteryear (about a decade or more ago) and ambiguous, and authors only had to comply with three clauses (i.e., the fourth existing clause did not exist then). The issue of authorship verification is currently critical following the use of large language models and artificial intelligence-driven chatbots, like ChatGPT, which do not meet ICMJE clauses of authorship ^(3)^.

However, updating these recommendations may present pitfalls for journals and authors adjusting their practices to comply. For example, the ICMJE transitioned its conflicts of interest (COI) form template to a “disclosure form” in February 2021, formalizing it in June 2021, and the PDF version of the old form was replaced with a new form, in Word format, freely available from the ICMJE website (Figure 1). Two reasons were cited for this transition: 1) to reduce negative connotations and associations with the term “conflict,” and hence, the COIs document was rebranded as a “disclosure form”; 2) to expand the area of relationships and interests to include 13 possibilities ^(4)^. However, category #13 (“Other financial or nonfinancial interests”) is vague and leaves the accuracy of the form open to personal interpretation and bias, potentially invalidating the entire form. This risk is true for nonfinancial and/or personal COIs, which are often difficult to distinguish ^(5)^. Journals requiring authors to submit an ICMJE-based COI declaration must employ the new version because it is an ethics-related statement and a legal document. In addition, journals must be coherent. Therefore, if they claim to adhere to the ICMJE recommendations, they must enforce such policies. The ease with which authors can complete the current ICMJE disclosure form should not distract them from the caveats associated with unclear clause #13. In such a situation, it is best to consult with the journal and editor-in-chief before submission. Meanwhile, journals must ensure that ICMJE disclosure form is up-to-date (see examples in Table 1 where the old form is still in use), and that their ethical guidelines and policies are consistent to avoid confusion and errors on the part of authors who may feel misguided.

**Figure 1.**
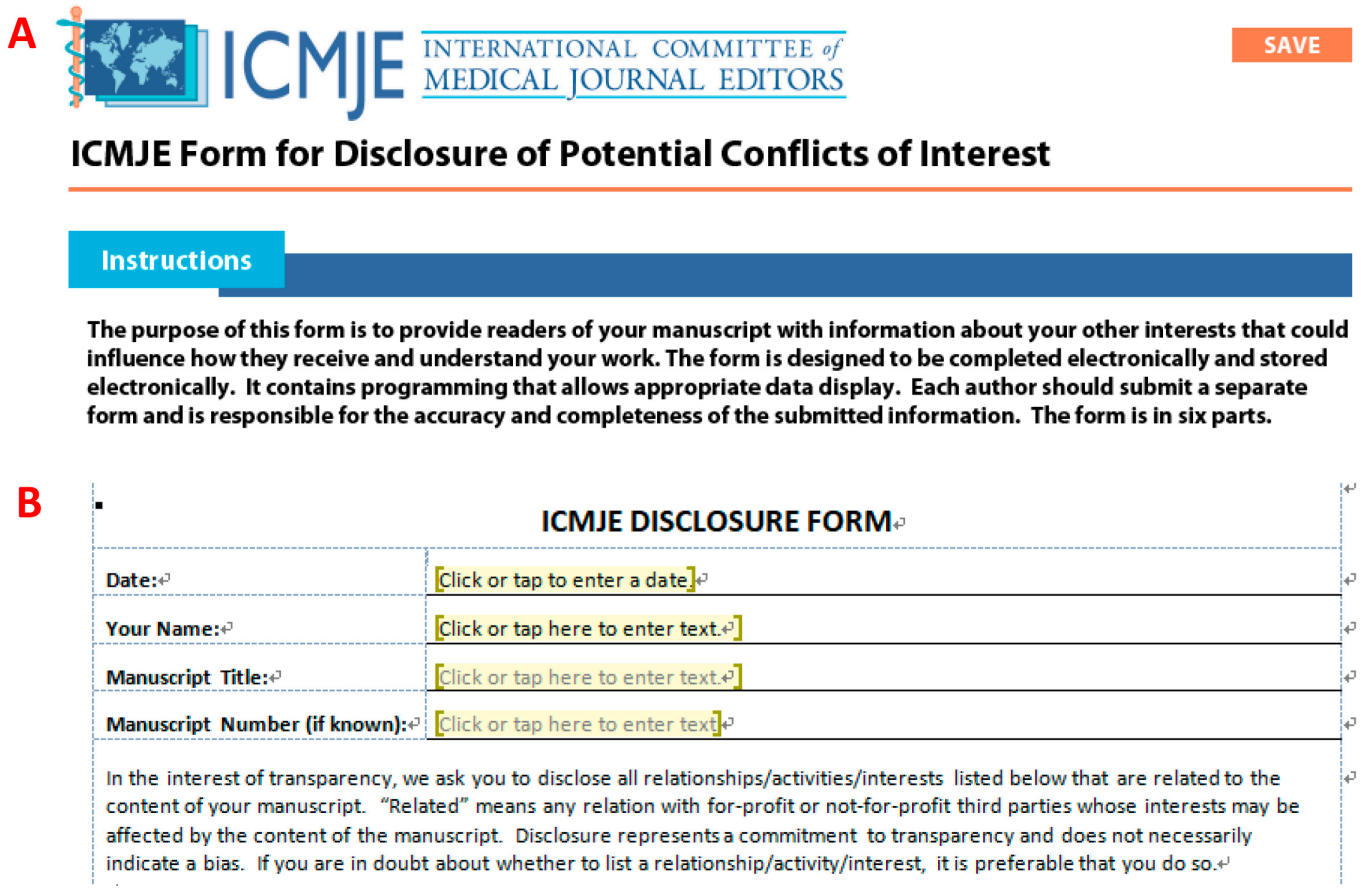
Screenshot of the top section of the old (until about August 2021) ICMJE COI (A) and the current disclosure form (B). Fair use for academic purposes (A).

**Table 1.** Examples^1^ from Four Publishers Using the Old ICMJE COI Form Rather than the Updated ICMJE Disclosure Form.

Journal	Publisher	Relevant URL(s)^2^
*Journal of Health Psychology*	SAGE	https://journals.sagepub.com/author-instructions/HPQ#Authorship https://journals.sagepub.com/pb-assets/cmscontent/HPQ/coi_disclosure.pdf
All journals	Baishideng Publishing Group	https://www.wjgnet.com/bpg/GerInfo/287 https://f6publishing.blob.core.windows.net/customuploadedfiles/Conflict-of-interest_statement.pdf
*Value in Health*	International Society for Pharmacoeconomics and Outcomes Research, Inc.	https://www.ispor.org/publications/journals/value-in-health/for-authors/guide-for-authors https://www.ispor.org/docs/default-source/value-in-health/conflict-of-interest-form-2022.pdf
*Journal of Applied Pharmaceutical Science*	Open Science Publishers LLP	https://japsonline.com/policies.php?id=11 https://japsonline.com/admin/php/uploads/3677_pdf.pdf

^1^ Nonexhaustive list discovered on Google in a search on June 21, 2023 ^2^ Where possible, relevant URLs were archived at the Internet Archive (https://archive.org/web/), as irrefutable historical proof

In their opinion paper, Matsubara and Takahashi ^(1)^ offered some excellent ideas to residency specialists, who would also benefit from knowing the information in this opinion. Crucially, they must check the ICMJE recommendations for updates, just in case some policies concerning them have been updated. They must also check journals’ websites carefully to ensure ICMJE compliance. If they find a non-ICMJE-compliant journal, they should be cautious about submitting work to that journal.

Finally, we wish to raise a rarely debated issue, namely the retrospective application of current ICMJE recommendations to older papers. For example, for any reason, if there is an ethical investigation by a journal into a paper’s research, should the 2022 version of the ICMJE recommendations be applied to older papers, say from 2017 or 2007? Evidently not, and authors of such investigations must ensure that journals present the version of the ICMJE recommendations that existed at that time. Despite this logic, we have no evidence that current rules and guidelines are being retrospectively applied.

Therefore, practicing the claims made within academic publishing is vital for the integrity of science. Editors claiming to follow ICMJE recommendations have an ethical obligation to follow and implement updates made to them. Authors should consider the truthfulness of claims made by editors as a criterion for deciding on where to publish their research. More generally, academics must intricately reflect on the dynamic nature of publication ethics because of its inextricable and growing impact on research careers.

## Article Information

### Conflicts of Interest

None

### Author Contributions

The authors contributed equally to this paper’s intellectual discussion, literature exploration, writing, reviewing, and editing.

### Approval by Institutional Review Board (IRB)

Not applicable.

### Informed Consent

Not applicable.

### Patient Anonymity

Not applicable.
